# Polyethylene Glycol (PEG)-Induced Anaphylaxis: A Cautionary Case for the Gastroenterologist in the COVID Era

**DOI:** 10.7759/cureus.93953

**Published:** 2025-10-06

**Authors:** Maziar Fazel Darbandi, Branden Thorsteinson, Alexandra Ilnyckyj

**Affiliations:** 1 Department of Surgery, University of Manitoba, Winnipeg, CAN; 2 Department of Anesthesiology, University of Manitoba, Winnipeg, CAN; 3 Department of Internal Medicine, University of Manitoba, Winnipeg, CAN

**Keywords:** adult-onset allergy, allergy and anaphylaxis, gastroenterology and endoscopy, laxative, polyethylene glycol

## Abstract

Polyethylene glycol (PEG) has widespread use and, while initially thought to be inert, has proven to be immunogenic. We discuss the case of a 37-year-old female patient who was admitted to the intensive care unit (ICU) after sustaining a cardiac arrest in the community. Fifteen minutes after ingestion of PEG, she developed hand swelling, which over minutes progressed to difficulty with breathing and swallowing. The Allergy service was consulted. Anaphylaxis was suspected by the presentation and confirmed after detailed exposure and ingestion history, along with an acute tryptase rise. Allergy to PEG was confirmed by skin testing in the outpatient allergy clinic after the patient was discharged from the hospital.

## Introduction

Polyethylene glycol (PEG) is widely known for its common use as an over-the-counter laxative. However, it also has widespread use as an excipient in pharmaceuticals, an emulsifier or lubricant in cosmetics, and an additive in food products [1]. It has proven to be an excellent bulking and stabilizing agent, as the covalent attachment of PEG to these compounds increases their hydrodynamic size and increases their half-life [2]. Therefore, it is used as an excipient in many medications such as labetalol and acetaminophen [3]. Absorption of PEG molecules depends mainly on their molecular weight and mode of application [1]. For example, low-molecular-weight PEGs are more easily absorbed through the skin and gastrointestinal tract [1].

Initially thought to be non-immunogenic and a generally benign compound, more recent evidence has proven otherwise [2,4]. Garay et al. reported that anti-PEG antibodies have been found in the blood of 25% of healthy blood donors, proving that PEG indeed has immunogenic effects [5]. Most reactions to PEG-containing compounds are elicited by high-molecular-weight PEGs, such as macrogol bowel preparations [6,7]. Interestingly, PEG was also used as an excipient in the Pfizer-BioNTech and Moderna vaccines for COVID-19 [7-12]. The COVID vaccines differ from previous common vaccines in this regard [7-12]. This was thought to be a factor in the immunogenicity of these vaccines compared to previously engineered vaccines [7-12].

Case reports and series have been published describing PEG-induced allergic reactions and anaphylaxis [7-12]. Unfortunately, the evidence is limited, and larger population epidemiological data are not available to describe PEG-induced allergy, further emphasizing it as a rare occurrence. It has been proven that PEG-induced anaphylaxis occurs via an IgE-mediated type 1 hypersensitivity reaction [2].

## Case presentation

A 37-year-old female patient was admitted to the intensive care unit (ICU) after sustaining a cardiac arrest in the community. The patient had an uneventful birth by C-section. She developed post-operative constipation. She had treated constipation effectively three years earlier with over-the-counter PEG. She tolerated the product well and decided to use it again. Fifteen minutes after ingestion, she developed hand swelling, which over minutes progressed to difficulty with breathing and swallowing, and urticarial rash. Paramedics were called and found the patient to be hypotensive and hypoxic. Based on the history, paramedics suspected anaphylaxis and treated the patient with a total of 5 mg of epinephrine, given intramuscularly, while transporting her to the hospital. During this time, she demonstrated pulseless electrical activity. Resuscitation continued as she was transported to the hospital. Return of systemic circulation (ROSC) was achieved in the hospital 45 minutes after her cardiac arrest. After ROSC, she developed rebound symptoms of diffuse erythema and hypotension, resulting in further epinephrine therapy and the addition of solumedrol and diphenhydramine, which proved effective.

The Allergy service was consulted. Anaphylaxis was suspected by the presentation and confirmed after detailed exposure and ingestion history, as well as an acute tryptase rise (34 mcg/L on presentation) with subsequent normalization (1 mcg/L 6 days later). The Allergy service documented four potential drug exposures: (1) PEG 15 minutes before circulatory collapse; (2) labetalol, which she had been taking regularly three times per day for several days; (3) acetaminophen, which she had taken in the hospital after her cesarian section and led to a rash and hives, leading to her not taking it anymore; and (4) COVID vaccination: three doses of the Pfizer BNT162b vaccine with the most recent one occurring about three weeks before her presentation to the hospital. Skin testing was not pursued early due to the risk of anergy and negative testing. Delayed testing could not be pursued due to the patient’s clinical deterioration with intercurrent health problems, including *Clostridioides difficile* colitis, sepsis, and pancreatic trauma from the LUCAS-3 CPR device. The temporal relationship between the ingestion of PEG and the onset of symptoms made PEG the most likely culprit. Months later, after she had eventually been discharged from the hospital, she underwent skin testing with appropriate controls, which confirmed the allergy to PEG.

## Discussion

PEG’s widespread use in a variety of compounds can make tracking exposure and possible sensitization difficult. Among the other case reports and series published describing PEG-induced allergic reactions, our patient is similar in demographics when compared to the other patients [7-12]. Also, her anaphylactic reaction was incited by a high-molecular-weight PEG compound, in keeping with our current understanding of PEG-induced allergy and anaphylaxis [6,7].

In our case, the patient developed a rash and hives after taking acetaminophen for pain after her cesarean section. Acetaminophen is a PEG-containing compound, and this intolerance may have hinted at sensitization to PEG-containing medications [1,3,4,7]. However, physicians may still see this class of products as inert due to their frequent and over-the-counter use. This case highlights the devastating consequences of having a low index of suspicion for PEG intolerance.

PEG is widely used in endoscopy for bowel preparation [1]. Although the reaction is rare and there are no specific guidelines as to how to screen patients before using PEG, it would be wise for the gastroenterologist or endoscopist to be aware that patients can develop allergies to PEG [1-6]. If a patient reports allergic-type symptoms during PEG use or during the use of other PEG-containing compounds, the symptoms should not be dismissed as intolerances; rather, an allergy to PEG should be considered.

## Conclusions

This case provides an important lesson on the devastating consequences of an allergy to PEG. In the era of COVID and the growing use of PEG as an excipient in vaccines, the modern physician should keep the possibility of allergy to PEG at the forefront of their mind. Patients can be referred to an allergist for assessment before the elective use of PEG-containing products again. If urgent use is required, then observation/premedication should be considered.
